# Rare imaging phenotype of late gadolinium enhancement in a patient with anoctamin 5 mutation

**DOI:** 10.1093/ehjcr/ytae626

**Published:** 2024-11-23

**Authors:** Qi Zhang, Jinghui Li, Minjie Lu

**Affiliations:** Department of Magnetic Resonance Imaging, Fuwai Hospital and National Center for Cardiovascular Diseases, Chinese Academy of Medical Sciences and Peking Union Medical College, Beilishi Road No. 167, Xicheng District, Beijing 100037, China; Department of Radiology, First Hospital of Shanxi Medical University, Jiefang South Road No. 85, Taiyuan 030001, Shanxi, China; Department of Magnetic Resonance Imaging, Fuwai Hospital and National Center for Cardiovascular Diseases, Chinese Academy of Medical Sciences and Peking Union Medical College, Beilishi Road No. 167, Xicheng District, Beijing 100037, China; Department of Magnetic Resonance Imaging, Fuwai Hospital and National Center for Cardiovascular Diseases, Chinese Academy of Medical Sciences and Peking Union Medical College, Beilishi Road No. 167, Xicheng District, Beijing 100037, China

## Case summary

Anoctamin 5 (ANO5) is a putative intra-cellular calcium-activated chloride channel. Recessive mutations in ANO5 most commonly present as muscular dystrophy, with cardiac involvement rarely reported. Here, we report the case of a 55-year-old woman who initially presented with symptoms of cardiac abnormalities. Cardiac magnetic resonance imaging (MRI) revealed dilated cardiomyopathy, characterized by a rare late gadolinium enhancement (LGE) pattern mimicking ischaemic LGE, despite normal coronary arteries.

## Case presentation

**Figure 1:**
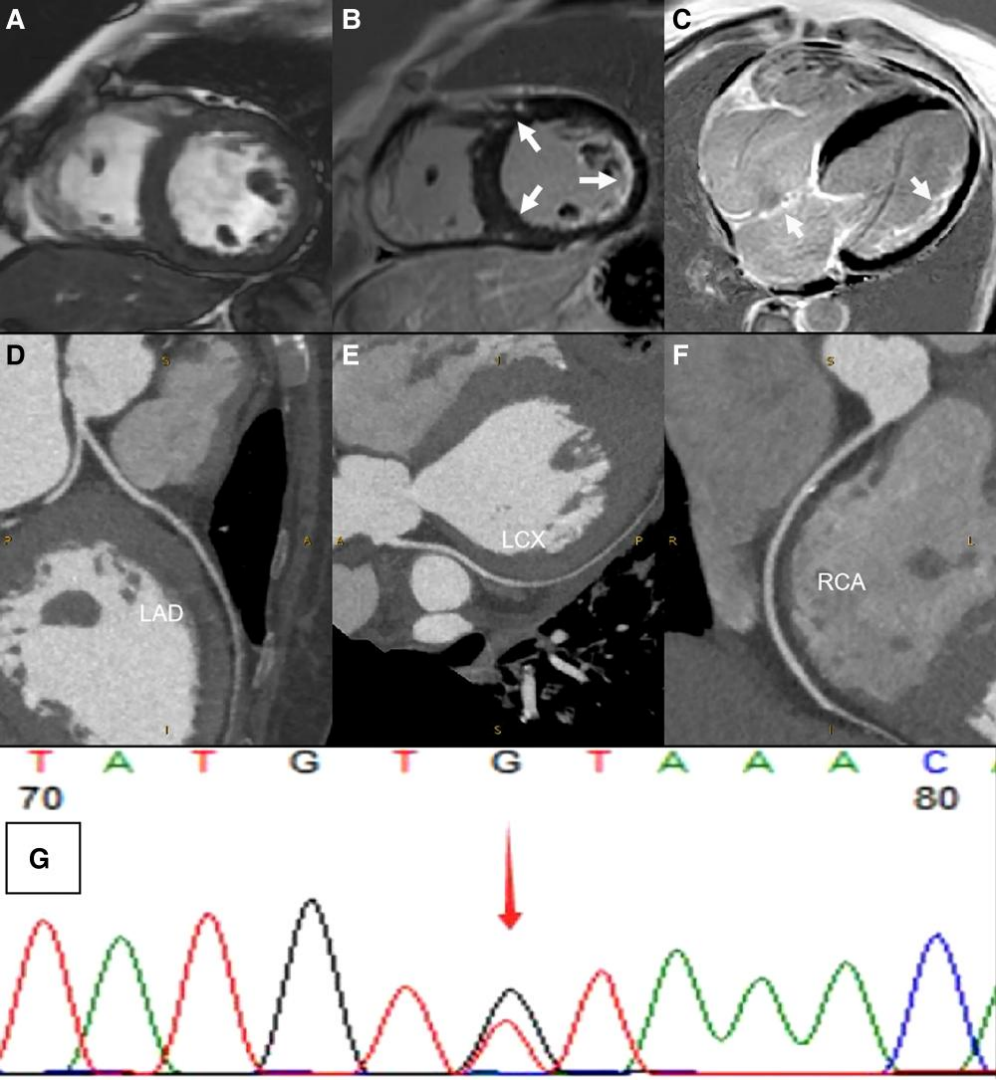
(*A*) Cardiac magnetic resonance imaging short-axis sagittal view showing a moderately dilated left atrium and ventricle, with mild inter-ventricular septum thickening (15 mm). (*B* and *C*) Late gadolinium enhancement at 30 min post-contrast showed a region of enhancement at the right ventricular insertion points, sub-endocardium of the left ventricular lateral wall, trabeculae, and inter-atrial septum (arrow). (*D–F*) Coronary computed tomography angiography showed the left anterior descending artery, left circumflex coronary artery, and right coronary artery with no significant stenosis. (*G*) Whole-exome sequencing identified the mutation (anoctamin 5 c.1301G>T).

A 55-year-old female experienced intermittent dyspnoea, palpitations, and chest tightness over 10 years. Her 24-h Holter monitor revealed frequent premature ventricular contractions, sinus pauses, and second-degree sinoatrial block. Laboratory tests showed elevated N-terminal pro b-type natriuretic peptide (2893 pg/mL; normal range <150 pg/mL) and creatine kinase (239 IU/L; normal range 0–200 IU/L). Cardiac MRI indicated enlargement of the left atrium and ventricle, mild inter-ventricular septum thickening (15 mm), and reduced left ventricular systolic function with an ejection fraction of 26% (*Figure 1A*). Late gadolinium enhancement was present in the right ventricular insertion points, sub-endocardium of the left ventricular lateral wall, trabeculae, and inter-atrial septum (*Figure 1B* and *C*). Coronary CT angiography showed no significant stenosis (*Figure 1D–F*). A biopsy from the right ventricular septum showed non-specific findings. Despite no muscular symptoms, muscle biopsy pathology showed necrotic myopathic changes without myositis antibodies. Whole-exome sequencing identified an ANO5 gene mutation (c.1301G>T, *Figure 1G*). ANO5 encodes anoctamin-5, a putative intra-cellular calcium-activated chloride channel, highly expressed in skeletal and cardiac muscle and bone tissues. Some studies indicate that dilated cardiomyopathy with ANO5-related myopathy can present with intra-myocardial LGE and are often clinically asymptomatic cardiac-wise.^1,2^ In this case, the patient’s initial manifestation was cardiac involvement. As the ANO5-related LGE located in the right ventricular insertions and lateral wall has not been previously reported, this case underscores the importance of cardiac MRI and genetic testing in diagnosing ANO5-related dilated cardiomyopathy, revealing unique imaging patterns that expand our understanding of ANO5-associated cardiac conditions and highlights the need for advanced diagnostic and personalized treatment strategies.

## Data Availability

There were no new data created or analysed in this study.

## Notes

**Consent:** The authors confirm that written consent for submission and publication of this case report, including images and associated text, has been obtained from the patient in line with COPE guidance.

**Funding:** This study was funded by the Construction Research Project of Key Laboratory of Chinese Academy of Medical Sciences (grant no. 2019PT310025), the Youth Key Program of High-level Hospital Clinical Research (grant no. 2022-GSP-QZ-5), and Beijing Natural Science Foundation (7242110).
